# Self-management of type 2 diabetes among Turkish immigrants in Norway: A focus group study

**DOI:** 10.1177/22799036231154680

**Published:** 2023-02-08

**Authors:** Betül Cokluk, Miroslava Tokovska

**Affiliations:** Department of Health and Exercise, School of Health Sciences, Kristiania University College, Oslo, Norway

**Keywords:** Diabetes, Turkish immigrants, Norway, health literacy, focus groups

## Abstract

The prevalence of Type 2 Diabetes Mellitus (T2DM) is higher among Turkish immigrants than the general population in Norway. The aim of the study is to describe the challenges and experiences faced by Turkish immigrants in Norway in the self-management of T2DM. The study design is based on descriptive research using a qualitative approach. The sample group contained 13 persons participating in three focus group interviews: nine women and four men. A phenomenological-hermeneutical approach was employed to achieve a deeper understanding of the experience of self-management of T2DM among Turkish immigrants in Norway with regard to HL. The participants described experiences of the T2DM self-management with regard to HL and revealed three major themes: (1) understanding the role and responsibility of health care staff in T2DM treatment, (2) assessing T2DM education course and information and (3) applying knowledge and motivation to adapt to life with T2DM. Findings from this study revealed that self-management of patients with T2DM among Turkish immigrants is related to their cultural, religious and socio-economical background and experiences. By understanding the cultural features, a well-tailored intervention according to the needs of Turkish immigrants regarding self-management can be developed. Health care staff are recommended to consider patients’ HL when interventions are developed.

## Introduction

The prevalence of type 2 diabetes is high and rising across the world.^1^ There are inequalities in the prevalence of T2DM in Europe; minorities develop T2DM at an earlier age than the host European population.^2,3^ T2DM in Norway is diagnosed up to 15 years earlier in first-generation immigrants from Asia.^4^ Research studies from Denmark, Austria, the Netherlands and Norway show that the prevalence of T2DM is higher among immigrants.^4,5,6^ Additionally, morbidity and mortality due to T2DM among immigrant groups in Norway are more common.^7^

Self-management of T2DM is an essential part of treatment and is dependent on patient motivation and ability to increase physical activity and develop healthy dietary habits.^8^ People with TD2M were shown to have levels of health literacy (HL) associated with higher levels of education, better overall health conditions and higher self-perceived empowerment. No empirical evidence strengthening either the link between HL and glycaemic control or the link between HL behaviours was found.^9^ The Norwegian government defines HL as *‘a person’s ability to understand, assess and apply health information enabling them to make knowledge-based decisions related to their health. This applies to decisions related to lifestyle choices, disease prevention measures, self-management of disease and use of health and care services’.*^10^ In 2019 the Norwegian government, emphasised the prevalence of low HL among immigrants and their corresponding knowledge of chronic diseases. This led The Norwegian Directorate of Health, under the commission of The Ministry of Health and Care Services, to launch a strategy aimed at strengthening HL within the target group(s). HL is a clear significant factor in diabetes self-management, with several research studies indicating an association between HL and empowerment.^11–13^ HL is composed of different types of literacy in hierarchical classification^14^:

• *Basic/functional literacy;* the lowest level, indicating sufficient basic skills in reading and writing to be able to function effectively in everyday situations.
• *Communicative/interactive literacy; more* advanced cognitive and literacy skills enable persons with this level of HL, together with their social skills, to actively participate in everyday activities, extract information and derive meaning from different forms of communication, and apply new information to changing circumstances.
• *Critical literacy;* the most advanced cognitive skills. This type of HL can, together with social skills, be applied to critically analyse information, and to use the information to exert greater control over situational events.^14^

According to the Health Literacy Population Survey (2019), a higher proportion of the Turkish group scored lower within specific datasets with regard to HL, such as the health promotion domain. This domain includes finding, understanding, appraising and applying health information in a health promotion context.^6^

Previous studies on T2DM showed that Turkish immigrants in European countries have difficulties due to motivation, poor adherence to medication, and irregular health routines.^15,16^ Turkish immigrants in European countries expressed difficulties in treatment and self-treatment due to differences in culture and religion in the host country.^8,15,16^ In general, individuals with poorer HL, lower levels of social support and more severe depression also have worse diabetes outcomes.^17^ According to Statistics Norway, 18.5% of the population in Norway can be classified with an immigrant background, with Turkish immigrant groups being one of the largest non-Western groups since the 1970s.^18^ Although a high prevalence of T2DM was identified among Turkish immigrants in Norway, there are no studies investigating the difficulties faced by Turkish patients during diagnosis and treatment, and the impacts of HL in self-managing T2DM.

Health coaching (HC), a patient-centred approach to disease management, is an effective intervention and approach to improving health behaviours and reducing the global burden of chronic diseases, including diabetes.^19^ Results from studies about patients with T2DM receiving HC as a TD2M self-management strategy reveal self-efficiency of T2DM self-management, including blood sugar control and a healthy diet.^20–22^

We will thus describe the challenges and experiences faced by Turkish immigrants in Norway in the self-management of T2DM. The main research question of this study is: *What challenges and experiences do Turkish immigrants in Norway have with self-management of T2DM?*

## Design and methods

This study used a qualitative descriptive research design. A phenomenological-hermeneutical approach was employed to achieve a deeper understanding of Turkish immigrants’ experience with self-managing T2DM in Norway with regards to HL. The methodology offers a unique opportunity to record individuals’ subjective experiences and interpret a phenomenon within a specific context of care.^23^ Hermeneutics is based on the understanding that the formation of knowledge includes a form of interpretation. The chosen methodology helps in interpreting the meanings discovered or adds value to those type of interpretations. The researchers are conscious of and reflective about the ways in which their questions, methods and subject position might impact on the data produced in a study.^24^

Data were primarily collected from focus group interviews with providers, enabling a proper understanding of the experiences of Turkish immigrants that have managed T2DM on their own and with consideration of their HL.

## Setting and sample

The participants recruited for this study consisted of a selected sample of four men and nine women with T2DM living in Norway. The participants were divided into three focus groups; the first consisting of four women, the second four men and the third five women.

The reason behind the choice of gender-based interviews is that gender role may play a role in lifestyle changes,^8,25^ and females experience sociocultural changes differently from men. The way power is distributed in most societies means that women have less access to and control over resources to protect their health and are less likely to be involved in decision-making. Gender based approach also reveals health risks and problems which women face as a result of the social construction of their roles. Further, a gender-based approach should be implemented for health evaluations.^15,26^

The participants were recruited through Turkish mosques and social media groups. On November 23, 2021, a mosque was visited by the researcher (BC) to provide information about the project and to recruit participants through a snowball sampling technique for two of the three focus groups. Snowball sampling is a method that has been extensively adopted in focus group research, especially when it is related to vulnerable and marginalised participants that are difficult to reach.^27^ The first focus group was at the home of one of the participants, while the two others were held at an office of a volunteer centre. The participants who were invited to the interview chose to get the information about the study in Turkish. The interviews were conducted by the author in Turkish. This was due to the participants’ lack of fluency in Norwegian and their ability to express themselves more properly in their native tongue.

The inclusion criteria included being diagnosed with T2DM, being an immigrant from Turkey, and being able to conduct the interview in either Norwegian or Turkish. The age range of the participants was 41–71 years old, with an average age of 56.6 years old. In addition, the participants all emigrated from Turkey and have lived in Norway either most or all their adult lives. The mean residence time was 35.5 years. Two of the 13 participants were illiterate, one had a university degree and the rest had an education of 11 years (high school level).

Each focus group discussion started with an introduction to the purpose and content of the study, while participants were asked to give informed consent (written or oral form). All participants gave informed, oral consent and anonymity was ensured by transcribing the interviews to safeguard privacy.

## Ethical approval

The protocol of the study and related documents were approved by the Norwegian Centre for Research Data (NSD) (Ref. 108907 approved on the 25th of October 2021).

## Data collection

The research data were obtained by focus group interviews using a semi-structured form. The interview guide was translated into Turkish by the author and piloted by two persons with Turkish backgrounds. A pilot interview is an interview conducted to give the interviewer some experience with the questions and imbue them with a greater sense of confidence.^28^ All data were collected in an urban area in southeast Norway and interviews were performed between November-December 2021. The interviews were recorded on digital devices and lasted between 29 and 69 min, with an average of 53 min.

A semi-structured interview guide was used, starting with open-ended questions. This qualitative study is based on HL theory. The interview guide is HL-focused, and the interview findings have been discussed with respect to HL theory and previous similar findings for T2DM patients. The open-ended questions were designed to elicit spontaneous discussion concerning each person’s experience in self-managing T2DM with regards to HL. The interviewer aimed to achieve an atmosphere of respect and trust by inviting the participants to openly describe their experiences as part of the phenomenological-hermeneutical approach.^19,23^ The questions for the focus groups centred on the participants’ practices and experiences related to their diagnosis of T2DM. The content of the interview was predicated on HL theory with the results of the studies conducted on T2DM and immigration, together with observations made among the Turkish participants. The questions asked in the study were about their experiences with diabetes education courses, motivation and knowledge of T2DM. Examples of the final questions are:

• Tell us about your experiences in connection with diabetes education courses. Have you been offered a diabetes education course?
• What motivates you to adapt your life to T2DM?
• Do you know of any negative effects or complications associated with T2DM? If you do, please describe them.

The interviews were first transcribed in Turkish by the author and then translated to English. All transcripts were reviewed many times to eliminate any errors and then checked for accuracy against the audiotapes.

## Data analysis

Data analysis was carried out by the phenomenological-hermeneutical approach.^29^ The research method included three steps: (1) naive reading, (2) structural thematic analysis and (3) thorough understanding.^29^

The first arc of interpretation is the naive reading. The naive interpretation is the initial interpretation of the full and the main random interpretation of the impact of the researcher’s pre-understandings. The aim was to understand the interview texts as a whole and to understand the meaning of the phenomenon. The second arc of interpretation, the ‘structural analysis’, focuses on explaining the phenomena. A thematic structural analysis was applied with the aid of a reflective distance, enabling us to better condense the meaning units within the text and create themes and sub-themes. The whole text was examined and divided into units. The themes were validated back and forth in relation to the naïve understanding, resulting in improved naive understanding. The third arc of interpretation, the ‘comprehensive understanding’, is characterised by a critical synthesis of the evolving results. To get a deeper understanding of the phenomenon and its meaning, the critical synthesis is interpreted in relation to other texts, such as previous research findings and philosophical texts. In this last phase, the in-depth interpretation was primarily based on naive understanding, thematic structural analysis, prior research knowledge and the theoretical context. The result is presented following the three arcs of interpretation.

## Results

The Turkish immigrants that participated in the three focus groups described their experiences with self-managing T2DM. The analysis showed that the results are shedding light on (1) Understanding the role and responsibility of health care staff in T2DM treatment, (2) assessing T2DM education courses and information and (3) applying knowledge and motivation in the ability to adapt to life with T2DM.

### Understanding the role and responsibility of health care staff in T2DM treatment

All the participants had knowledge and interest in T2DM, and many reported some complications that can occur or had occurred, including diseases in the eyes, kidneys and feet. The general practitioner (GP) was the main source of information for most of the participants, as shown in the following participant’s views:

*‘Actually, they (the GPs) are doing what they are supposed to. [But] we don’t’. (P1) ‘. . .they are doing what is necessary (. . .) we don’t apply what the doctor said’. (P4)*

*‘I think my doctor is doing what he has to do*’. *(P5)*

Other participants (P6-P9 and P12) shared an overall satisfaction with their GPs and emphasised:

*‘They help on a regular basis, answer our questions (. . .) So I am personally satisfied!’*

None of the participants specified their GP’s ethnic background. Some of the participants (P10, P11 and P13) discussed negative aspects with their GPs, including difficulties with insufficient information and guidance and not being taken seriously as explained in the following interview:

*‘Sometimes doctors don’t give a full explanation, it’s like leaving it to the patient themself’. (P13)*

*‘They never sent me to the polyclinic or anywhere similar (. . .) I never got that kind of help’. (P11)*

*‘When you wake up in Turkey, there is sun in the morning. But when you get up here, you can’t see the sun for a week, it also has an effect’. (P8)*

Other participants noted and referred to changes in habits, as quoted in the interview:

*‘We say we should not worry about anything, but when we hear something, it doesn’t go away’. (P4)*

*‘People are always on the move here, but I guess it’s because of stress’. (P11)*

A variety of emotions were described, such as stress, sadness, fear and nervousness, as stated below:

*‘My biggest fear is, if I’ve had my condition since childhood then how is my kidney now? How is my liver? How are my eyes?’* Another participant (P4) reported: *‘It (T2DM) neither kills you nor gets better. It makes you suffer. (. . .) There is sugar in everything. I am confused about what to eat’. (P10)*

### Assessing diabetes education courses and information

None of the 13 participants was offered a course on diabetes education when they were diagnosed with T2DM. P10 and P11 emphasised the lack and a need for this course and shared the frustration as follow:

*‘At the general practitioner*, *the diagnosis was made, then he wrote (the prescription for) the injection (. . .) I bought it from the pharmacy, the girl there said a few words (about how to use the medicine). I came home and used it. I never got any other kind of help. I don’t know any more, was it necessary to go to the hospital, to receive such a diabetes education course? They (the GP) never offered anything like that’. (P11)*

As a substitute for a lack of education courses participants usually try to find more relevant health information about T2DM from other sources, including from newspapers, social media, Turkish television programmes, family, friends, brochures and the Norwegian Diabetes Association. The health information and help are not tailored to immigrant patients as stated in the interview

*‘*When it isn’t explained properly, you just do what you think is best (. . .) people *need to be given advice that is closer to their own culture and language’. (P10)*

Participants P5, P8 and P13 expressed a preference to utilise health care services and health information from Turkish healthcare personnel in Turkey as quoted in the interview

*‘. . .You feel more secure (in Turkey). You’re saying, here’s a Turkish doctor, let me pour it out, completely* (. . .) *My nephew is a dietitian in Istanbul (. . .) I ask him, and he sends me things monthly’. (P5)*

*‘I always go both here and in Turkey (. . .) There is no queue in Turkey, it’s quick, the system is good, there’s no problem if you have money’. (P8)*

*‘My cousin’s daughter is a doctor in Turkey. She told me to watch my sugar (T2DM)’. (P13)*

On the other hand, a male participant reported his distrust of the health care system in Turkey:

*‘I don’t trust Turkey as much as you do. More precisely, for some reason, as far as possible, I prefer the health sector in Norway rather than Turkey*’. *(P7)*

Participants from all three focus groups elaborated on their experiences that being diagnosed with and living with T2DM is emotionally difficult, even though they receive knowledge-based information about their disease and treatment. Several participants linked some of the negative emotions and why they were diagnosed with T2DM with their background as migrants as reported:

*‘So we all came from our country to another country. We experienced traumas, but we lived them without knowing it was trauma. And of course, eating habits. I also gained weight after that. I gained a lot of weight’. (P13)*

*‘There is also the effect of our being in a foreign country (. . .) We cannot be involved in family matters when it is necessary to go to Turkey or when we need to go. We’re obsessing over this’. (P7)*

All 13 participants preferred to receive health information in Turkish, even though many emphasised that they knew Norwegian.

### Apply knowledge and motivation in the ability to adapt to life with T2DM

The participants expressed difficulties in adapting their lives to T2DM. In all three focus groups, the lifestyle changes in terms of diet were discussed. All the participants expressed the importance of healthy diets and said they had made dietary changes.

*‘I’m trying not to eat bread for breakfast (. . .) Vegetables, green peppers, red peppers, tomatoes, a piece of cheese, eggs’.* Another (P5) illustrated: *‘Same dish, same thing, but mine is a small plate (. . .) My wife prepares my daily things. Even for work*’. Another participant (P8) noted: *‘My wife cooks normally’. (P13)*

One of the important sources of difficulties is Turkish cuisine, as eating homemade and traditional dishes was described as an important part of their cultural identity and connection with their family members. But it was difficult for some of the participants to be the only ones with T2DM in the family as commented from the interviews:

*‘You have to isolate yourself’*. (P10)

*‘I cook my own food’.* (P6)

*‘There is sugar in everything. I am confused about what to eat’.* (P4)

Further, the participants shared some of their common thoughts on Turkish hospitality as one participant stated:

*‘*. . . .*obligation (to eat a host’s food) is in our culture. . .’* (P5)

Other participants shared the unhelpful behaviour on the part of the host, with participants (P9 and P13) reporting hosts’ comments, such as:

*‘Eat this! Nothing will happen’.*

Three of the four male participants pointed to their wife’s role in cooking and care and the important role of food in the culture of hospitality. Despite this, one of the participants emphasised:

*‘I know how to say “no” when I am a guest (. . .) I’m comfortable with that’. (P6)*

Participants tried to adapt their life to T2DM because they have concerns about the illness:

*‘It (T2DM) [affects] our eyes, kidneys. . .We’re just trying to [not make it worse]. There can be wounds on the foot, God forbid, that do not heal. [Then] the foot [has to be] cut off’. (P4)*

*‘I pay great attention to the kidneys’*. (P6 and P8)

Some of the women stated that it is difficult to fully pay attention to themselves because of their role in the family as one participant illustrated:

*‘I can’t fully pay attention to myself’. (P1)*

The female participants added that they were responsible for preparing food for their families and they were reluctant to prepare more than one dish at a time, a healthy dish for themselves and a traditional dish for the rest of the family as shared:

*‘It’s good when I am alone in the house [and don’t need to prepare more dishes]’. (P13)*

*‘In fact, if people were left alone (. . .) then they would more pay attention (to themselves). For example, in my case’. (P10)*

Regarding the motivation to adapt their lives to T2DM, the participants had different reflections: Three of the four men reported their job as a motivational factor.

*‘It is work that motivates. In order not to lose the job. I’m doing my best’. (P6)*

The importance of movement was highlighted too.

*‘Movement helps. My sugar is high when I don’t go (to exercise). It falls when I go. I’m glad’. (P4)*

## Discussion

The participants expressed a boundary to Turkish cuisine culture and many of them to the health care system in Turkey. These findings confirm the argument that the most difficult thing to manage for the Turkish immigrants in these health conditions is the food habit part of their identities.^15^ The Norwegian Directorate of Health (2021) has a statutory mandate to publish national professional guidelines, advice and recommendation to the health services and population related to diabetes.^30^ According to this guideline, all newly diagnosed patients with diabetes have the right to a good education, for example through a start-up education course. This is also legislated as a part of the Specialist Health Services Act in Norwegian legislation, which came into force in 2001.^31^ The GP must inform the patient and offer a referral to such a course at the nearest hospital. The Norwegian Directorate of Health (2021) states that the specialist health service has the responsibility to offer diabetes education courses in groups. The course usually takes place under the auspices of the Learning and Mastery Centres. This course is an addition to continuous individual follow-ups under the auspices of the interdisciplinary competence team (diabetes team).^30^ It is worrying that none of the 13 participants in this study has been offered this service.

A Dutch study about diabetes education among Turkish immigrants with T2DM suggests that Turkish patients with a longer history of TD2M are more difficult to motivate when it comes to completing an education programme. They also observed a relationship between dropouts and their poor knowledge of T2DM.^32^ Therefore, it is important to receive an education on diabetes when the condition is newly diagnosed. This gives the patient the opportunity of assessing the service, regardless of their HL. The association of HL with self-management behaviours in patients with T2DM revealed that diabetes education was effective in improving self-management, diabetes knowledge and blood sugar control – regardless of the level of HL.^33^ They pointed out that expanding educational programmes for patients with low literacy may reduce differences in diabetes outcomes related to literacy status. More tailored educational programmes for patients with low literacy and HL should be under consideration in the health care system.^6^ A HC programme would be an opportunity to have the patient in focus and take the patient’s socioeconomic, cultural and religious background into account when adapting the treatment programme to the patient or adapting an existing programme to the patient.^33^

Participants are exposed to information about T2DM through several channels. The Turkish immigrants find it easier than the majority of the population to appraise whether the mass media’s information on health risks is reliable, and they have a high critical HL; at the same time, it is more difficult for the Turkish immigrants to communicate with health care professionals.^6^ Even though the participants were, overall, satisfied with their GP and health care staff and the information they get in Norway, many of them used a second medical opinion in Turkey. This approach shows that the participants have trust in the Turkish health care service and some unmet needs in the health care system in Norway. Additionally, all of them prefer to get health information in Turkish. This indicates that the participants have strong boundaries to their homeland and culture. The participants were actively involved in their disease and treatment, but many of them extract and assess information both from Norwegian and Turkish health care staff. Relating to two guidelines for T2DM from two different countries can lead to confusion and stress as to whether the information will be understood, assessed and applied in their daily self-management of T2DM.

Given that the participants emigrated mostly from rural areas in Turkey to urban areas in Norway, their food habits changed considerably in the host country. The food in Norway is overall richer and higher in energy.^34^ Even if the participants want to take care of their diet and eat diabetes-friendly, it is difficult to be the one in the family who has diabetes. In Turkish culture, it is important for the family to eat together, and many participants expressed that they felt excluded when they ate different dishes to their families. As previous qualitative studies show, self-management of T2DM is emotionally, physically and socially challenging for immigrants.^15,35^ Eating the traditional dishes of their home country was described as an important part of their cultural identity and more than just a means of sating hunger. The support of family is important for individuals with T2DM; family members are a key source for assisting and encouraging dietary changes and as such they should be invited to partake in an educational programme.

People are expected to participate in health decisions and take responsibility for their own health despite more complicated health problems and the need to navigate a more complex health system. A previous study in Norway showed that health care professionals should tailor health information to the individual’s HL level and educational level. Furthermore, they should be prepared to devote more time to explaining relevant health information, using different learning aids and ensuring that the individual thoroughly understands the information.^9^ It is essential to meet the patient on their level of HL in a health care system where the individual is the key person for critically analysing information and employing the information necessary for adapting to life with T2DM.

Shared decision-making between the patient and health care professional is a keyphrase in the health care system in Norway. Participation in health decisions and the right to health information is legislated as a part of the Patient and User Rights Act in Norwegian legislation, which came into force in 2001.^36^ This study has illustrated that T2DM patients with a Turkish immigrant background can be active participants in health examinations and consultations, not just passive recipients, when more consideration is paid to their needs.

## Practice implications

T2DM is a complex chronic condition that requires healthy living habits, including diet, physical activity, self-management of medication, and often lifestyle changes. The study results reveal the importance of considering HL levels when developing health information and programmes.

Findings from the study indicate the unmet needs of Turkish immigrants in the Norwegian healthcare system. Diabetes education courses should be organised to develop T2DM awareness, not only among the patients but also involving family members as useful channels of health information. It would be more effective if a campaign targetting such individuals could be adapted culturally and linguistically. Healthcare staff and family members must understand and consider the emotional burden of living with T2DM as an immigrant in Norway.

## Strength and limitations

This study has several limitations. Firstly, many of the participants were recruited through mosques and hence their religious lifestyle should be considered in T2DM self-management and their approach to their disease. Secondly, only one of the authors collected data on the diabetic self-management experiences of chosen focus groups at a single point in time; hence, the results may not accurately reflect any changes in an individual’s perspective over time. Furthermore, in interviews conducted in languages other than Norwegian or English, the analysis was based on a transcription of one of the researchers like as translator’s words, which may not always adequately represent the exact wording used by participants. This was part of the reason why the thematic structural analysis methodology was chosen to avoid an over-reliance on the semantics of translated expressions.

The main strength of our study is that one of the researchers (BC) can speak the immigrants’ language and knows the sociocultural environment and traditions. As a woman of Turkish descent, author BC shared certain identities with the participants. Some participants made references to themselves and author BC as ‘us Turkish people’ or noted that that author BC was around the age of their own children and made references such as ‘my daughter’. For instance, the first group interview at the house of one of the participants was extended to an afternoon tea with snacks. However, this position may have also constrained participants towards considering and discussing their Turkish immigrant identity, which is common in research studies like this.^8,15,25,35^ This study benefits from the direct narrative accounts of Turkish immigrants, who were very diverse in terms of their age, gender, education, health conditions, place of residence and length of stay. Even though the research team consisted of two individuals with different professional backgrounds and qualitative research, this study represents unrepeatable stimuli for further research into the issue of chronic diseases among other groups of migrants.

Future studies should involve patients from a range of settings to ensure that our findings represent a larger cross-section of Turkish immigrants.

## Conclusion

Findings from this study revealed that challenges and experience with self-management of patients with T2DM is related to their socioeconomic, cultural and religious background and experiences. Understanding these cultural features can lead to a well-tailored intervention based on the needs of Turkish immigrants regarding disease self-management. Healthcare staff are recommended to consider the patient’s HL when interventions are developed. Both healthcare staff and family members must understand and consider the emotional burden of living with T2DM as an immigrant in Norway. One of the possible opportunities for increasing HL and self-management of patients with T2DM could be to offer a HC programme in the Turkish language, provided by healthcare staff with a Turkish background.

In order to meet the need for information, individualised educational programmes should be considered for future effective education health care interventions of T2DM and HC in Norway. Finding from this study may help decision-makers, health care professionals and civil society to understand the complexity of challenges faced by Turkish immigrants in Norway in self-management of chronic disease as Type 2 diabetes mellitus.

## References

[1] World Health Organization. Diabetes, https://www.who.int/news-room/fact-sheets/detail/diabetes (2021, accessed 4 February 2022).

[2] Agyemang C van der Linden EL Bennet L . Type 2 diabetes burden among migrants in Europe: unravelling the causal pathways. Diabetologia 2021; 64: 2665–2675. 34657183 10.1007/s00125-021-05586-1 PMC8563673

[3] Sat S Aydinkoc-Tuzucu K Beger F , et al. Diabetes and migration, German Diabetes Association: Clinical Practice Guidelines. Diabetologie 2020; 15(1): 232–248.

[4] Jenum AK Diep LM Holmboe-Ottesen G , et al. Diabetes susceptibility in ethnic minority groups from Turkey, Vietnam, Sri Lanka and Pakistan compared with Norwegians - the association with adiposity is strongest for ethnic minority women. BMC Public Health 2012; 12(1): 150. 22380873 10.1186/1471-2458-12-150 PMC3315409

[5] Kristensen JK Bak JF Wittrup I , et al. Diabetes prevalence and quality of diabetes care among Lebanese or Turkish immigrants compared to a native Danish population. Prim Care Diabetes 2007; 1(3): 159–165. 18632038 10.1016/j.pcd.2007.07.007

[6] The Norwegian Directorate of Health. Health literacy in five immigrant populations in Norway: Pakistan, Poland, Somalia, Turkey, and Vietnam, https://www.helsedirektoratet.no/rapporter/befolkningens-helsekompetanse/.pdf (2021, accessed 28 February 2022).

[7] Kjøllesdal M . (Red). Helse blant innvandrere i Norge. Levekårsundersøkelse blant innvandrere 2016. Norwegian Institute of Public Health, https://www.fhi.no/publ/2019/helse-blant-innvandrere-i-norge-levekarsundersokelse-blant-innvandrere-2016/ (2019, accessed 14 March 2022).

[8] Abuelmagd W Håkonsen H Mahmood KQU , et al. Living with diabetes: personal interviews with Pakistani women in Norway. J Immigr Minor Health 2018; 20: 848–853. 28698971 10.1007/s10903-017-0622-4

[9] Finbråten HS Guttersrud Ø Nordström G , et al. Explaining variance in health literacy among people with type 2 diabetes: the association between health literacy and health behaviour and empowerment. BMC Public Health 2020; 20: 161. 32013897 10.1186/s12889-020-8274-z PMC6998369

[10] Norwegian Government. Strategi for å øke helsekompetansen I befolkningen 2019-2023, https://www.regjeringen.no/no/dokumenter/strategi-for-a-oke-helsekompetansen-i-befolkningen-2019-2023/id2644707/ (2019, accessed 16 January 2022).

[11] Abdullah A Liew SM Salim H , et al. Prevalence of limited health literacy among patients with type 2 diabetes mellitus: A systematic review. PLoS One 2019; 14(5): e0216402. 10.1371/journal.pone.0216402 PMC6504081 31063470

[12] Lee E-H Lee YW Lee K-W , et al. A new comprehensive diabetes health literacy scale: Development and psychometric evaluation. Int J Nurs Stud 2018; 88: 1–8. 30142483 10.1016/j.ijnurstu.2018.08.002

[13] Shin KS Lee EH . Relationships of health literacy to self-care behaviors in people with diabetes aged 60 and above: empowerment as a mediator. J Adv Nurs 2018; 74(10): 2363–2372. 29893030 10.1111/jan.13738

[14] Nutbeam D . Health literacy as a public health goal: a challenge for contemporary health education and communication strategies into the 21st century. Health Promot Int 2000; 15: 259–267.

[15] Biyikli Gültekin E . Difficulties in health care for female Turkish immigrants with type 2 diabetes: a qualitative study in Vienna. Wien Klin Wochenschr 2017; 129: 337–344. 28382526 10.1007/s00508-017-1190-2

[16] Peeters B Van Tongelen I Duran Z , et al. Understanding medication adherence among patients of Turkish descent with type 2 diabetes: a qualitative study. Ethn Health 2015; 20(1): 87–105. 24588791 10.1080/13557858.2014.890174

[17] Moskowitz D Thom DH Hessler D , et al. Peer coaching to improve diabetes self-management: which patients benefit most? J Gen Intern Med 2013; 28: 938–942. 23404203 10.1007/s11606-013-2367-7 PMC3682027

[18] Statistics Norway. Immigrants ang immigrant’s descendants (in Norwegian), https://www.ssb.no/befolkning/statistikker/innvbef (2021, accessed 27 January 2022).

[19] Wolever RQ Simmons LA Sforzo GA , et al. A systematic review of the literature on health and wellness coaching: defining a key behavioral intervention in healthcare. Glob Adv Health Med 2013; 2(4): 38–57. 10.7453/gahmj.2013.042 PMC3833550 24416684

[20] Chen RY Huang LC Su CT , et al. Effectiveness of short-term health coaching on diabetes control and self-management efficacy: a quasi-experimental trial. Public Health Front 2019; 7: 314. 10.3389/fpubh.2019.00314 PMC6831637 31737593

[21] Howard LM Hagen BF . Experiences of persons with type II diabetes receiving health coaching: an exploratory qualitative study. Educ Health (Abingdom) 2012; 25(1): 66–69. 10.4103/1357-6283.99210 23787387

[22] Pirbaglou M Katz J Motamed M , et al. Personal health coaching as a type 2 diabetes mellitus self-management strategy: a systematic review and meta-analysis of randomized controlled trials. Am J Health Promot 2018; 32(7): 1613–1626. 29658286 10.1177/0890117118758234

[23] Creswell JW Poth CN . Qualitative inquiry and research design: choosing among five approaches. London: SAGE publications, 2017.

[24] Sloan A Bowe B . Phenomenology and hermeneutic phenomenology: the philosophy, the methodologies, and using hermeneutic phenomenology to investigate lecturers’ experiences of curriculum design. Qual Quant 2014; 48: 1291–1303.

[25] Rieger EY Terragni L Czapka EA . Experiences and perceptions of body weight among Turkish immigrant women in Norway. Int J Migr Health Soc Care 2021; 17(1): 92–104. 2021.

[26] World Health Organization. Gender analysis in health: a review of selected tools, https://apps.who.int/iris/bitstream/handle/10665/42600/9241590408.pdf;jsessionid=0AF7A155F61070B4A3373C70F9D5F1DB?sequence=1 (2002, accessed 15 December 2022)

[27] Liamputtong P . Focus group methodology. Principles and practice. Singapore: SAGE publications Asia-Pacific Pte Ltd, 2012.

[28] Espedal G Løvaas B Sirris J S and Wæraas, S. Researching Values. Methodological approaches for understanding values, understanding values. Work in organisations and leadership. London: Palgrave Macmillan, 2022.

[29] Lindseth A Norberg A . A phenomenological hermeneutical method for researching lived experience. Scand J Caring Sci 2004; 18(2): 145–153. 15147477 10.1111/j.1471-6712.2004.00258.x

[30] The Norwegian Directorate of Health. Diabetes, https://www.helsedirektoratet.no/retningslinjer/diabetes (2021, accessed 28 February 2022).

[31] Norwegian Legislation. Lov om spesialisthelsetjenesten m.m, https://lovdata.no/dokument/NL/lov/1999-07-02-61/KAPITTEL_3#%C2%A73-3 (2022, accessed 18 January 2022).

[32] Uitewaal P Hoes A Thomas S . Diabetes education on Turkish immigrant diabetics: predictors of compliance. Patient Educ Couns 2005; 57: 158–161. 15911188 10.1016/j.pec.2004.05.009

[33] Kim S Love F Quistberg DA , et al. Association of health literacy with self-management behavior in patients with diabetes. Diabetes Care 2004; 27(12): 2980–2982. 15562219 10.2337/diacare.27.12.2980

[34] Aambø A . Innvandreres helse og helsetjenestens ansvar. Oslo: Cappelendamm, 2021.

[35] Abuelmagd W Osman BB Håkonsen H , et al. Experiences of Kurdish immigrants with the management of type 2 diabetes: a qualitative study from Norway. Scand J Prim Health Care 2019; 37: 345–352. 31299877 10.1080/02813432.2019.1639911 PMC6713117

[36] Norwegian Legislation. Lov om pasient- og brukerrettigheter, https://lovdata.no/dokument/NL/lov/1999-07-02-63 (2022, accessed 18 January 2022).

